# Enhancing public health surveillance: a comparative study of platform-specific and hybrid assembly approaches in SARS-CoV-2 genome sequencing

**DOI:** 10.1099/mgen.0.001357

**Published:** 2025-07-10

**Authors:** Yasemin Coşgun, Süleyman Yalçın, Ege Dedeoğlu, Gültekin Ünal, Katharina Kopp, Biran Musul, Ekrem Sağtaş, Philomena Raftery, Gülay Korukluoğlu, Sedat Kaygusuz

**Affiliations:** 1 The National Virology Reference Laboratory, Public Health General Directorate, Ministry of Health, Ankara, Türkiye; 2 The Department of National Reference Laboratories and Biological Products, Public Health General Directorate, Ministry of Health, Ankara, Türkiye; 3 Public Health General Directorate, Ministry of Health, Ankara, Türkiye; 4 The National Molecular Microbiology Reference Laboratory, Public Health General Directorate, Ministry of Health, Ankara, Türkiye; 5 World Health Organization Country Office in Türkiye, Ankara, Türkiye; 6 World Health Organization Country Office in Türkiye, Ankara, Türkiye, from January 2022 to May 2023; 7 Department of Pathobiology, School of Veterinary Medicine, St. George’s University, True Blue, Grenada, West Indies; 8 Department of Medical Microbiology, University of Life Sciences, Ankara City Hospital, Ankara, Türkiye

**Keywords:** genomic surveillance, hybrid assembly, Illumina, Oxford Nanopore Technologies, SARS-CoV-2

## Abstract

During the COVID-19 pandemic, next-generation sequencing (NGS) has been instrumental for public health laboratories in tracking severe acute respiratory syndrome coronavirus 2 (SARS-CoV-2) mutations and informing responses. Illumina systems and Oxford Nanopore Technologies (ONT) have been primary tools for NGS, each presenting unique advantages. The hybrid assembly (HA) approach, integrating short- and long-read sequencing methods, has been developed to improve genome accuracy by utilizing the combined advantages of both techniques. While HA has been used to enhance SARS-CoV-2 genome quality, its optimal applications for SARS-CoV-2 sequencing and surveillance have not been systematically studied. This study seeks to address this gap by evaluating the conditions under which HA improves SARS-CoV-2 genomic surveillance, analysing 192 samples using eight bioinformatics methods across both platforms. HA was evaluated against single-technology approaches for its genome assembly and mutation detection performance. While HA did not outperform single-technology methods in detecting unique mutations, it produced marginally more complete genomes than Illumina-based methods. Importantly, mutations identified by HA were consistently detected across all eight methodologies, demonstrating its reliability in mutation detection. Moreover, our research underlines the critical need for in-house validation of methods and exposes the limitations inherent in proprietary pipelines. Our findings suggest that an HA approach could be used as a quality control tool in genomic surveillance, particularly for improving low-quality ONT sequencing data by integrating high-quality Illumina sequencing data. However, implementing HA demands the presence of both sequencing platforms and additional resources, such as hands-on time, expensive sequencing reagents and bioinformatics know-how. A decision-tree analysis identified the percentage of trimmed ONT reads relative to total reads as crucial for HA success, emphasizing the significance of high-quality ONT reads. This comprehensive approach provides public health laboratories insights to refine genomic surveillance strategies for SARS-CoV-2, potentially influencing future research and response efforts.

**Impact Statement**

This study explores the hybrid assembly’s role in enhancing COVID-19 genomic surveillance by analysing 192 routine surveillance samples with eight bioinformatics methodologies. We uncover conditions where hybrid assembly significantly improves genome completeness, especially for samples processed by Oxford Nanopore Technologies with extensive trimming. This advancement offers public health labs an incremental yet crucial tool for analysing challenging COVID-19 samples. The research also emphasizes the difficulties public health laboratories face due to the limited access to proprietary sequencing data, such as primer sequences, to conduct in-house bioinformatic analysis. It underscores the need for standardized laboratory and bioinformatics practices, suggesting that clearer guidelines and open methodologies could enhance the reliability and accessibility of genomic surveillance. By highlighting both the potential of hybrid assembly in specific scenarios and calling for increased transparency and standardization in genomic surveillance, the study contributes an important increment to the field, with implications for improving public health responses to COVID-19.

## Data Summary

The authors confirm all supporting data, code and protocols were provided within the article or through supplementary data files.

All Illumina and ONT sequencing reads were submitted to the ENA under study PRJEB73346.

All the R scripts utilized to create the figures in this article were submitted to GitHub.

(github.com/EgeDede562/hybrid_assembly_comparison and github.com/EgeDede562/COVID19_Inhouse_Script)

## Introduction

Severe acute respiratory syndrome coronavirus 2 (SARS-CoV-2), the causative agent of the COVID-19 pandemic, had been considered a public health emergency of international concern for more than 3 years starting from January 2020 to March 2023 [1,2]. During this period, the pandemic has resulted in over 760 million cases worldwide and more than 6.9 million deaths [3].

Studying the genetic variations in SARS-CoV-2 across cases and regions has become a critical tool in the fight against COVID-19. This approach enables scientists to track mutations and identify variants of concern (VOC), which can influence containment strategies and vaccine development. Additionally, analysing the phenotypic qualities of these variants, such as increased virulence or resistance, can affect the global scientific community and public health response. Such collaborative efforts have deepened our understanding of the dynamics of the pandemic and fostered global collaboration.

Genomic surveillance has played a critical role in managing the pandemic, facilitating the development of diagnostics, antivirals and vaccines and enabling transmission tracking between communities [4,6]. The first genome of the SARS-CoV-2 virus was uploaded to international databases within days of its detection [7]. Access to this initial genome has enabled global research efforts to identify mutational changes in the virus through sequencing [8,9]. This has led to tracking its spread [10], identifying transmission clusters, guiding contact tracing, informing intervention strategies [11,12] and influencing vaccine and therapeutic developments [13,15].

Whole-genome sequencing (WGS) of SARS-CoV-2 during the pandemic has been conducted chiefly using two commercial sequencing platforms, Illumina and Oxford Nanopore Technologies (ONT) [16,17]. Using short-read sequencing, Illumina is reliable for detecting single nucleotide polymorphisms. However, its limitations include challenges in assembling complex genome regions due to short read lengths [4,17, 18]. ONT offers longer read lengths, which can benefit *de novo* genome assemblies. Despite this advantage, ONT was found to have a relatively high error rate, which may have resulted in incomplete genome assemblies or lineage discrepancies [19], particularly when using R9 flow cells – the chemistry available during this study and much of the pandemic.

The effectiveness of genomic surveillance is closely tied to the accuracy of the genomes generated by these sequencing instruments and subsequent bioinformatics processes. For instance, errors in consensus genome sequences resulting from sequencing discrepancies or incomplete genomes can affect the accurate identification of VOCs or variants of interest. Such inaccuracies have implications for public health strategies. Recognizing these challenges, researchers have sought solutions to optimize the strengths of both sequencing methods [11,20, 21]. One such approach is hybrid genome assembly, which addresses gaps introduced by the consensus genome-building method and corrects potential errors by consolidating data from both read groups [22].

Hybrid assembly (HA) combines short- and long-read sequencing data to derive a comprehensive genome with potentially reduced errors [11]. Many hybrid assemblers have been developed to address incomplete genomes. These tools capitalize on the scaffolding capabilities of long reads and use short Illumina reads to fill in genomic gaps [23,25]. This approach has been used to identify specific regions of bacteria to increase confidence or completeness [26,27] and to identify different eukaryotic organisms [28,30].

HA has also been used to analyse SARS-CoV-2 in different contexts. Arana *et al*. previously utilized short and long reads to generate a hybrid consensus for 16 samples, including an RNA control. For the 15 samples, the hybrid method improved the percentage of mutations detected in the regions of the SARS-CoV-2 genome that were previously poorly covered [31]. Instead of using the widely used ARTIC primers [32] for long-read sequencing, they used in-house primers designed to generate long-read amplicons. Likewise, a study conducted in Mali used the hybrid approach, where researchers previously improved 11 poorly generated consensus genomes when first identifying SARS-CoV-2 in their country [33]. This allowed for a more detailed investigation of the first 21 cases in the country. However, it is important to note that these studies, primarily focused on refining sample quality and applying HA in routine SARS-CoV-2 sequencing, remain largely unexplored.

In December 2020, the National Reference Virology (NVRL) and National Molecular Microbiology Reference Laboratories (NMMRL) in Türkiye began employing Illumina and ONT for sequencing SARS-CoV-2 [34]. In October 2021, the NMMRL sequenced 192 samples using both technologies. This dual-technology sequencing aimed to explore any potential differences in lineage detection influenced by the sequencing platforms. This comparison focused on WHO nomenclature and lineage assignments, utilizing the proprietary analysis pipelines provided with the library preparation kits. At that time, the Delta variant of SARS-CoV-2 was predominant in Türkiye. The study found no differences in the lineage assignments between the samples sequenced by the two platforms, suggesting that Illumina and ONT had similar effectiveness in identifying SARS-CoV-2 lineages.

This study aims to systematically assess the utility of HA in SARS-CoV-2 genomic surveillance, exploring its capacity to enhance the accuracy and completeness of consensus genome results. By integrating short- and long-read sequencing technologies, we seek to elucidate the conditions under which HA could significantly contribute to the genomic surveillance of SARS-CoV-2, potentially offering insights into optimizing sequencing strategies for public health laboratories.

## Methods

### Sample collection, storage, nucleic acid extraction and library preparation

Through routine genomic surveillance, this study utilized nasopharyngeal swab samples obtained from individuals who tested positive for SARS-CoV-2 via quantitative PCR (qPCR) in Türkiye. Samples with a quantification cycle (*Cq*) value below 30 were selected for sequencing as reported by the referral laboratory. The NVRL received 192 samples on 6 October 2022. The NVRL received samples stored and preserved in vNAT® solution (Bioeksen R&D Technologies by the referral laboratory). Upon receipt, the samples were assigned a unique NVRL identification number (NVRL_ID) and kept at room temperature for 24 h while being preserved in vNAT®.

Nucleic acid extraction was performed the following day using the RINA M14 system (Bioeksen R&D Technologies), a magnetic bead-based automation system capable of handling 14 samples in 45 min, specifically from nasopharyngeal swabs in vNAT media, resulting in an 80 µl total nucleic acid solution.

The samples were stored in 96-well plates at +4 °C for 2 days after extraction before undergoing target enrichment and library preparation using CleanPlex SARS-CoV-2 Research Panels (Paragon Genomics). This process involved multiplex PCR reactions using 343 pairs of primers separated into two pools to cover the entire genome of SARS-CoV-2. Following this, they were kept at −20 °C until nanopore sequencing, 4 days after extraction. Nanopore sequencing libraries were generated using a modified version of the ‘Midnight Primers’ alongside the Rapid Barcoding Kit (SQK-RBK110.96, Oxford Nanopore Technologies) [35] [36]. Similarly, 11 µl of total nucleic acid solution was used for ONT library preparation, adhering to the ‘Midnight protocol’ specifications. As per the protocol, samples with a *Cq* value between 18 and 35 were processed without normalization [37]. Single amplicon library concentrations were measured but not recorded via the Qubit 4 fluorometer (Thermo Fisher Scientific) and were within the manufacturer’s suggestions, being at least 50 ng for both the Paragon kit and the Rapid Barcoding Kit. Individual libraries were not normalized before pooling to prepare the final libraries. Final libraries were prepared for both platforms by pooling 96 samples, resulting in two libraries per platform. For each pooled library, the library concentration was measured by the Qubit 4 fluorometer (Thermo Fisher Scientific). The pooled libraries concentrations were normalized to 8 pM for Illumina libraries per the manufacturer’s guidelines. For ONT sequencing, after rapid barcoding and assuming an average fragment size of 600 bp, the final library concentration was calculated to be ~210 pM, ensuring adequate pore occupancy during sequencing as suggested by the manufacturer [38]. The Illumina sequencing process took ~32 h on the MiSeq system in two batches of 96 samples each. For ONT, sequencing lasted 36 h on the GridION system, utilizing two flow cells simultaneously. Basecalling for the Illumina data was performed directly on the MiSeq instrument using bcl2fastq2 (v2.20). In contrast, using Guppy (v.5.0.14) with the High accuracy basecalling (HAC) model, basecalling for ONT data was conducted on the GridION. Sequencing data were acquired in fastq formats from Illumina and ONT for downstream analysis.

### Bioinformatics processes and data analysis

#### SARS-CoV-2 sample processing and consensus genome reconstruction using Illumina and ONT platform-specific sequencing data analysis pipelines

##### Consensus genome reconstruction

We obtained next-generation sequencing (NGS) raw reads from both Illumina and ONT sequencing platforms in fastq format, following demultiplexing and conversion from their primary formats (bcl for Illumina and fast5 for ONT) using each platform’s respective built-in workflows. We carried out initial quality assessments, variant calling and consensus genome reconstruction for the Illumina-derived samples using the SOPHiA DDM platform (version 5.10.43) designed for the Paragon CleanPlex kit-prepared samples [35]. In contrast, ONT sample analyses were performed using the proprietary software Massive Bioinformatics nCOV19 Analyser™ (version 1.0, Massive Bioinformatics R&D Technologies, 2021) [39].

Furthermore, we replicated the consensus genome reconstruction using the CoVpipe workflow (v0.2.7) [40], as implemented by the Robert Koch Institute for Illumina data and the ARTIC SARS-CoV-2 workflow (v0.3.15) [41] for ONT data, both of which were routine methods recommended and employed by public health institutions. These methods were used as reference benchmarks. For comprehensive analysis, our in-house script was also employed for all samples, ensuring platform-specific quality filtering, adapter trimming and primer removal using fastp [42] for Illumina samples (‘fastp --in1 input_R1.fastq --in2 input_R2.fastq --out1 output_R1.fastq --out2 output_R2.fastq --qualified_quality_phred 20 --length_required 50 --trim_poly_x --cut_tail --html report.html’). The ONT-derived FASTQ files were processed with NanoFilt (v.2.8.0) [43] to remove low-quality reads and adapter sequence trimming (-q 10 l 200 --maxlength 1300 --headcrop 10 --tailcrop 10). Subsequently, we conducted a quality control assessment of the processed reads from the Illumina and ONT platforms using FastQC (v.0.11.9) [44]. Mapping was conducted using bowtie2 (v.2.5.0) [45] for Illumina and minimap2 (v.2.24) [46] for ONT reads, both with default parameters, referencing the SARS-CoV-2 genome (GenBank entry NC_045512.2). Coverage and read mapping statistics were calculated, followed by consensus genome generation via iVar (v.1.3.1) consensus (‘iVar consensus -t 0.8 m 10 -q 30 r reference.fasta -b input.sorted.bam -o output_consensus.fa’) [47] for downstream applications, including phylogenetic analysis with NextStrain [48].

In addition to mapping-based approaches, we assembled whole genomes *de novo* using coronaSPAdes (SPAdes v.3.15.4) [49], enabling comparisons between reference-guided and *de novo* assembly methodologies.

##### Investigation of unclassified reads in ONT samples

Original raw FASTQ files were divided into two batches of 96 samples and processed using guppy_barcoder (version 6.5.7+ca6d6af) for additional barcode and adapter trimming. Both trimmed (guppy-processed) and original FASTQ files were quality filtered and quality controlled with NanoFilt and NanoPlot (v1.42.0), resulting in four datasets: original, original filtered with NanoFilt, guppy-trimmed and guppy-trimmed with NanoFilt. The filtered and unfiltered data from the original and guppy-processed datasets were analysed, and quality metrics were saved. Reads were classified taxonomically using Kraken2 (v.2.1.2) [50] with the standard database (Standard-8, Standard with DB capped at 8 GB), and both classification reports and unclassified reads were saved.

From the Kraken2 reports, the percentages of reads classified as *Betacoronavirus*, *Homo sapiens* and unclassified were analysed across three conditions: unfiltered (normal), processed with NanoFilt and trimmed with Guppy (guppy_barcoder). The elbow method was applied to the normal dataset using K-means clustering to determine the optimal number of clusters. The within-cluster sum of squares was calculated for one to ten clusters, and three clusters were chosen based on the ‘elbow’ point. K-means clustering (with three clusters) was performed on the normal dataset, and cluster centroids were generated. These centroids were then applied to the NanoFilt and Guppy datasets to assign samples to the closest corresponding cluster from the normal data, allowing for consistent comparison of clustering behaviour across all conditions. A parallel sets plot was used to visualize cluster shifts among the three conditions, illustrating each method’s sample transitions across clusters.

FASTQ files from unclassified nanopore sequencing reads were processed using a custom workflow with kmercountexact.sh (BBMap suite, version 39.08) [51] to extract k-mers. The top 500 k-mers from each file were selected based on abundance, using the parameters prefilter=10 and tossbrokenreads=t. This was conducted to analyse the unclassified reads to understand their composition. The parameters were used to ensure that only the most relevant k-mers that constituted were present within the unclassified reads were reported. K-mers were sorted, and the selected k-mers were saved as text files. Up to 700 unique k-mers from each directory were compiled into a master k-mer list. Both individual and master k-mer lists were converted to FASTA format. The master k-mers were subjected to blastn analysis (NCBI blast+version 2.9.0) [52] against a custom ONT rapid barcode database and the NCBI nt_core database (blastn -db nt -out ‘$blast_output’ -outfmt 6). Results were saved as text files for further analysis.

### HA of SARS-CoV-2 genomes from sequencing data generated by using Illumina and ONT systems

For the final assembly, reads from both platforms were combined for each sample. An HA approach was performed using hybridSPAdes (SPAdes v.3.15.4) [53], a tool designed to integrate sequencing data from short- and long-read technologies.

**Fig. 1.**
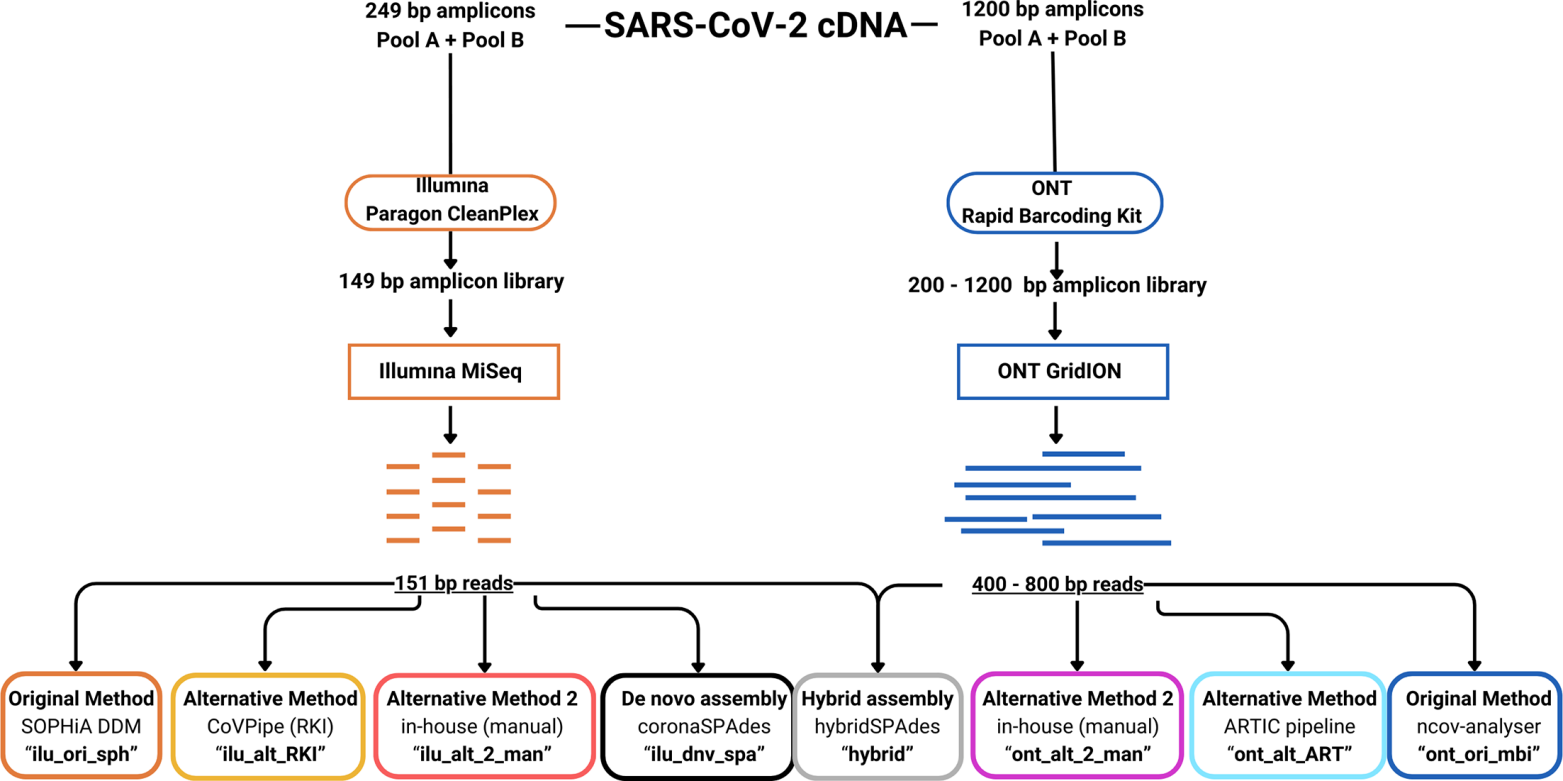
Summary of the bioinformatic pipelines used for this study. We used eight different analysis pipelines to analyse 192 samples. We used the SOPHiA DDM pipeline for Illumina systems to analyse 149 bp amplicons (ilu_ori_sph). In addition to SOPHiA DDM, we used Illumina reads for consensus genome generation using three additional pipelines: CoVPipe by the Robert Koch Institute (ilu_alt_RKI), our in-house method (ilu_alt_2_man) and *de novo* assembly using coronaSPAdes (ilu_dnv_spa). We used three approaches for ONT pipelines: the original proprietary pipeline (ont_ori_mbi), the ARTIC pipeline (ont_alt_ART) and our in-house script (ont_alt_2_man). Finally, we used hybridSPAdes (hybrid), which utilized both sets of reads for each.

A summary of the processes conducted can be seen in Fig. 1.

### Comparison of SARS-CoV-2 genome sequences using platform-specific workflows and hybrid approach

#### Quality assessment and lineage assignment with Nextclade

The SARS-CoV-2 genomic sequence quality was assessed, and lineages were assigned using Nextclade version 2.14.1 [54]. Comparison with a reference genome was done via Nextclade to analyse genome sequence length and mutation quantity, identifying sequencing errors and artefacts. This process ensured consensus genome standardization across different sequencing workflows. Lineages were also assigned to the sequences based on the Pangolin [55] and WHO nomenclature. All the Nextclade data were stored in an Excel file.

#### Data filtering for mutation analysis

An Excel file containing the percentage of genome covered (genome fraction) was extracted from the Nextclade files. A filter was created to compare the mutational profiles obtained from various methods on a per-sample basis. Prior to comparing mutational profiles obtained from different methods on a per-sample basis, a filtering step was applied to exclude samples with low genome fractions.

Samples were filtered based on genome coverage and method consistency. Only those with genome fractions >90 % in at least *n*−1 of the methods (where *n* is the number of methods providing genome fractions) were included for each sample. If a consensus genome was not generated for a sample by more than four methods, the sample was excluded entirely. This filtering process resulted in 95 ‘high-confidence samples’ for subsequent analysis.

#### Mutation data analysis and visualization

The results from Nextclade were used to generate a mutation dataset. The mutation data were organized with samples as rows and methods as columns, and the mutations detected in each sample were represented as a string value. The number of unique mutations in each sample was determined using R statistical software (v.4.3.2) [56] by comparing mutations detected using different methods. The unique mutations detected using each method were identified and reported. This process was performed for nucleotide substitutions, deletions and insertions and amino acid substitutions, deletions and insertions. To visualize the results, libraries ggplot2 (v.3.4.4) [57] and pheatmap (v. 1.0.12) [58] were used.

#### Categorization of mutations in ‘high-confidence samples’

The ‘high-confidence samples’ mutation dataset for amino acid substitutions was used for additional analysis. The data were imported into R using the readxl package (v.1.4.3) [59]. Mutations were binned based on their occurrence across different sequencing methods into the following categories: (1) unique (mutations observed only once across all methods), (2) universal (mutations observed in all eight methods), (3) high commonality (mutations observed in either six or seven methods), (4) medium commonality (mutations observed in either four or five methods) and (5) low commonality (mutations noted in either two or three methods). For each bin, the number of mutations was counted and reported.

#### Consensus matrix construction and method evaluation

Working with real-life surveillance samples, a conventional reference dataset could not be employed to identify mutations detected by the eight methodologies. A consensus matrix addressed this challenge as the operational standard for identifying ‘true positives’ (TP). This matrix was generated from the mutational data, wherein a mutation was included in the consensus set if detected by at least four of the eight different methods. The performance of each method was assessed using sensitivity and precision metrics calculated based on their respective TP, false positive (FP) and false negative (FN) counts. In brief, the sensitivity was calculated as TP/(TP+FN) and the precision was calculated as TP/(TP+FP). These values were visualized using the ggplot2 library in R.

### Data analysis of results from different bioinformatics pathways and the hybrid method

#### Calculating sample properties for the Illumina and ONT platforms

Taxonomical classification of sequencing reads, including distinguishing SARS-CoV-2 from other species or contaminants, was performed using ‘centrifuge’ (v.1.3.1) with default parameters and the compressed bacteria, archaea, viruses and human index [60]. This analysis provided two metrics: the total number of reads per sample and the number of SARS-CoV-2-specific reads per sample. From these, the percentage of SARS-CoV-2 reads in each sample was calculated as a third metric. The percentage of trimmed reads, calculated using fastp, was included as the fourth metric. Finally, a scoring method was developed from FastQC values by calculating the mean quality score for each base, referred to as the ‘General FastQC Score’, which served as a proxy for sample quality. These five metrics were collected for both sequencing platforms, resulting in ten metrics in total.

#### Descriptive statistical analysis

A comprehensive dataset was generated by collating the sample properties and metrics computed using eight methods. These metrics included genome fraction, number of amino acids (AA), nucleotide (nt) substitutions and deletions and the Nextclade quality score. Additionally, the dataset included the number of unique amino acid (AA) and nucleotide (nt) substitutions and unique nt and AA deletions. With eight methods employed, there were 80 columns of sample metrics.

Barplots were constructed and the normality of the 80 metrics was tested using the Shapiro–Wilk test [61]. A Kruskal–Wallis test [62] was performed to discern whether the metrics differed between the methods. If so, a post-hoc Dunn’s test [63] was conducted for each pairwise comparison. The *Z* values of each comparison were graphed, and heatmaps were generated for easier visualization.

### Classification analysis for hybrid variant detection

#### Logistic regression for hybrid variant detection

A logistic regression model was employed to predict the success of the hybrid variant detection method. The ‘glm’ function in R [64] was utilized to construct this model, specifying the binomial family. All sample properties from the Illumina and ONT platforms (*n*=10) were included as predictors to understand the probability of successful hybrid variant detection based on these characteristics.

#### Decision-tree classification

The decision-tree model was built using the rpart (v.4.1.21) package [65] in R, with the same predictors as in the logistic regression. The aim was to visualize and comprehend the decision nodes and primary splits that impact the hybrid detection success. The model’s performance was optimized by pruning fewer predictive branches, simplifying the decision tree.

#### Random forest analysis

A random forest classification model, comprising 100 decision trees, was constructed using the random forest (v.4.7–1.1) package [66] in R. To enhance the diversity and reduce the risk of overfitting, each tree was built with a random subset of three predictors at each split. The out-of-bag error rate was used to provide an unbiased estimate of the model’s accuracy, and its performance was evaluated on unseen data.

### Maximum-likelihood tree generation and representative sample selection

#### Sequence preparation and filtering

All consensus sequences obtained from each 192 for all eight methods were combined into a single multiple FASTA file. However, we filtered the combined FASTA files to ensure accurate alignment before tree generation. The initial processing of the sequence dataset involved two critical filtering criteria: the gap content and sequence length. Gap content, quantified by the proportion of undetermined nucleotides in a sequence, was limited to 20%. This threshold ensured the exclusion of sequences with a high amount of unknown or missing data. Additionally, sequences consisting of less than 90% of the reference genome (NC_045512.2, 29 903 bases) were excluded, setting a minimum acceptable length of 26 913 bases.

#### Maximum-likelihood tree generation

A phylogenetic tree was constructed using IQ-TREE (v.2.3.4) [67] with the HKY model based on 192 filtered SARS-CoV-2 sequences. Branch lengths, representing genetic distance as nucleotide substitutions per site, were evaluated, and branches showing less than 10^–7^ substitutions per site were collapsed into single nodes.

#### Downsampling and representative sequence selection

From an initial dataset of over 1300 SARS-CoV-2 sequences, we analysed sequences identified as hybrid variants (as determined by Nextclade analysis described above). Using the ape library (v.5.7–1) in R [68], we filtered for hybrid variant sequences based on their Nextclade lineage assignments, yielding 123 sequences. To identify representatives capturing maximum genetic diversity, we calculated pairwise genetic distances among all hybrid variant sequences using a custom implementation of the cophenetic function in R. From these distance calculations, we selected the 12 sequences with the highest pairwise genetic distances, representing ~10% of the hybrid variant subset.

#### Refining the phylogenetic tree

Following the selection of the representative sequences of hybrid variant sequences, their counterparts generated by all eight bioinformatics methods and the reference genome were included in the finalized tree. The final, refined phylogenetic tree was documented using the ‘write.tree’ function from the ape library.

## Results and figures

### Sample properties of 192 sequenced samples

**Fig. 2.**
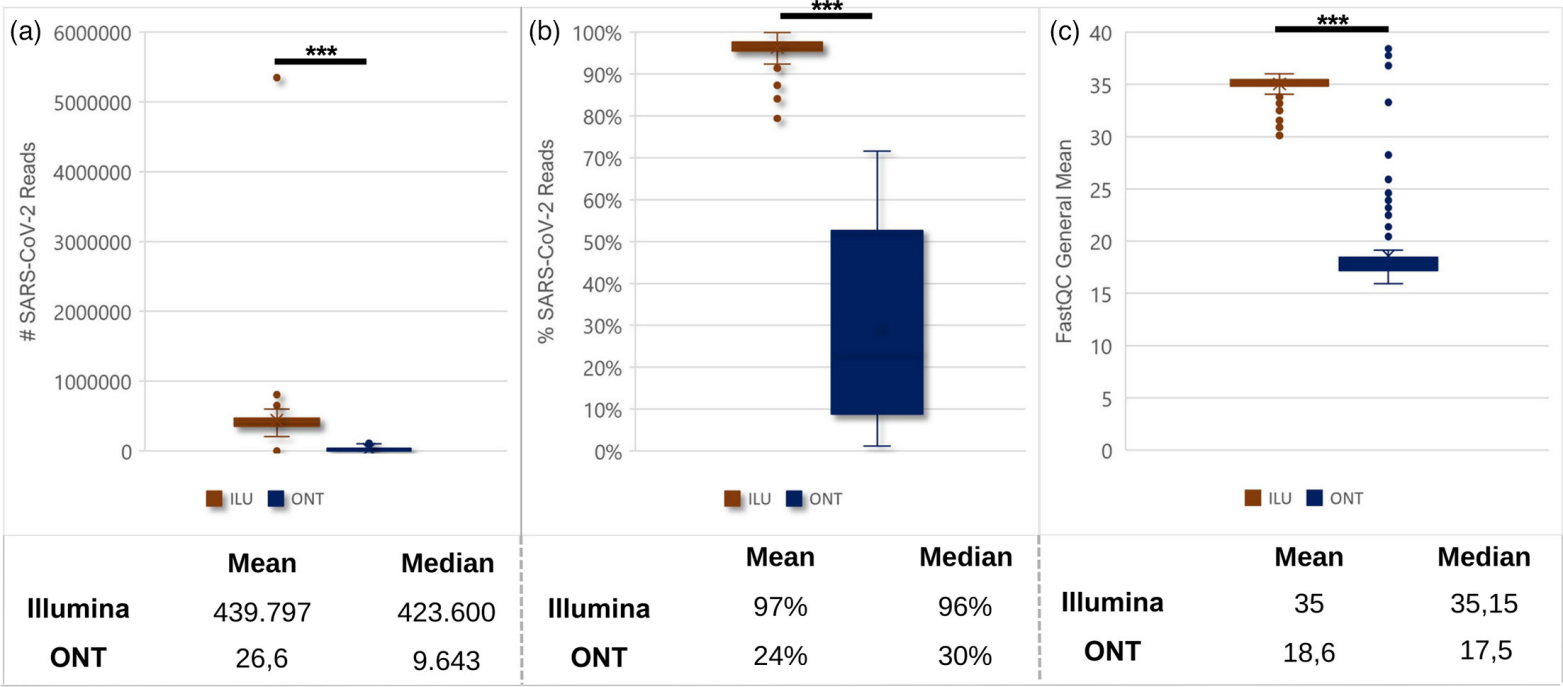
General overview of sample properties of 192 samples sequenced between platforms: comparison of sequencing metrics between Illumina (ILU) and ONT platforms for 192 SARS-CoV-2 samples. (**a**) Total SARS-CoV-2 read counts. (**b**) Percentage of SARS-CoV-2 reads relative to total reads. (**c**) FastQC general mean scores (average phred quality scores across all bases). Mean and median values are shown below each panel. Asterisks (***) indicate statistical significance (*P*<0.05).

The dataset comprised 192 SARS-CoV-2-positive samples collected during national genomic surveillance activities in Türkiye in early October 2022, before the Omicron variant was detected globally and when the Delta VOC (Delta) was the predominant circulating variant. Sequencing outputs were analysed across three parameters: SARS-CoV-2 read numbers, SARS-CoV-2 read abundance and base quality scores. The ONT platform generated 16.5-fold fewer SARS-CoV-2-specific reads compared to the Illumina platform (*P*<0.05) (Fig. 2a). The mean number of SARS-CoV-2 reads was 439 797 (median: 423 600) for Illumina and 26 600 (median: 9643) for ONT. The total input Illumina reads were >7.5× more than the ONT reads.

SARS-CoV-2 read abundance, calculated as the ratio of SARS-CoV-2 reads to total reads in each sample (expressed as a percentage), was determined to quantify the proportion of viral genetic material. Illumina-sequenced samples showed a mean SARS-CoV-2 read abundance of 97% (median: 96%). In contrast, ONT-sequenced samples exhibited a mean abundance of 24% (median: 30%), with ~25% showing abundances around 10%, indicating a higher presence of non-target genetic material in ONT-sequenced samples (Fig. 2b).

The FastQC general mean score, calculated as the average quality score for each base of every read within the sample, differed between platforms. Illumina samples showed a mean score of 35 (median: 35.15), while ONT samples showed a mean score of 18.6 (median: 17.5) (Fig. 2c). Analysis of non-SARS-CoV-2 reads in ONT samples revealed two categories: reads taxonomically classified as *Homo sapiens* and unclassified sequences (Table S1, available in the online Supplementary Material). To investigate whether unclassified reads originated from ONT barcodes or adapter sequences, samples were processed with guppy_barcoder, a tool of the Guppy software suite designed to detect and trim barcode and adapter sequences retrospectively from FASTQ files.

Filtering with NanoFilt reduced total read numbers while increasing the proportion of unclassified reads. Additional processing with Guppy for barcode trimming resulted in decreased SARS-CoV-2 read abundance. Filtering reduced the percentage of SARS-CoV-2 reads in each sample, and while the total number of reads decreased post-filtering, the relative proportion of unclassified reads increased. Guppy barcode removal did not raise the percentage of SARS-CoV-2 reads in filtered samples but further decreased SARS-CoV-2 abundance, with filtered short reads primarily originating from SARS-CoV-2 (Fig. S1a).

K-mer analysis (31 bp) of the 700 most abundant sequences in NanoFilt and guppy_barcoder-processed samples identified SARS-CoV-2-specific sequences among unclassified reads. In unprocessed samples, ONT barcodes and unclassified k-mers represented over 70% of total k-mers. Following guppy_barcoder processing, ONT barcode sequences and overall unclassified k-mers decreased, corresponding to an increase in SARS-CoV-2-specific k-mers (Fig. S1b). Some SARS-CoV-2 reads remained determined as unclassified even after barcode trimming.

### Comparison of different consensus genome generation algorithms alongside the hybrid method

We evaluated the performance of eight bioinformatic pipelines by analysing ten predefined metrics, as outlined in the Methods section. The complete descriptive statistical analysis is provided in Table S2.

### Method performance and lineage assignment across samples

Analysis of the 192 samples with Nextclade revealed that not all bioinformatics pipelines generated a consensus genome for every sample. Additionally, we observed that Nextclade failed to assign a lineage, or the lineage assignment would differ between methods even if a consensus is generated (Table 1). This analysis was limited to the samples classified as ‘detected’ by Nextclade.

**Table 1.** Lineage comparison of the 192 samples

WHO nomenclature	Sub-lineage	ILU ORI (SPH)	ILU ALT (RKI)	ILU ALT 2 (In-house)	ONT ORI (MBI)	ONT ALT (ARTIC)	ONT ALT2 (In-house)	ILU SPA (*de novo*)	Hybrid
**Delta**	21J	188	114	186	191	147	158	47	122
	21A	2	0	0	0	2	2	0	1
	21I	0	0	0	1	1	10	0	0
**Non-Delta**	19A	0	66	0	0	3	0	0	0
	20A	0	9	1	0	3	0	0	0
	Recombinant	1	0	0	0	18	0	0	0
**Total**		191	189	187	192	174	170	47	123

Most pipelines consistently assigned the Delta lineage, specifically the 21J sub-lineage, to over 90% of the genomes. However, lineage assignment varied depending on the pipeline. For instance, the Robert Koch Institute (RKI) pipeline (ILU ALT) identified a lower proportion of Delta lineages, with non-Delta lineages detected in over 60% of cases, associated with ambiguous bases recorded at lineage-defining mutation sites (Fig. S2a). Examination of the BAM files indicated that primer interference at mutation sites contributed to the ambiguity (Fig. S2b).

The ARTIC method (ONT ALT) identified 18 recombinant strains classified as Omicron–Delta recombinants (Table S3). Further examination of these samples showed low genome coverage and non-SARS-CoV-2 read abundance, indicating a misclassification by Nextclade due to a low-quality consensus genome obtained from this data.

**Fig. 3.**
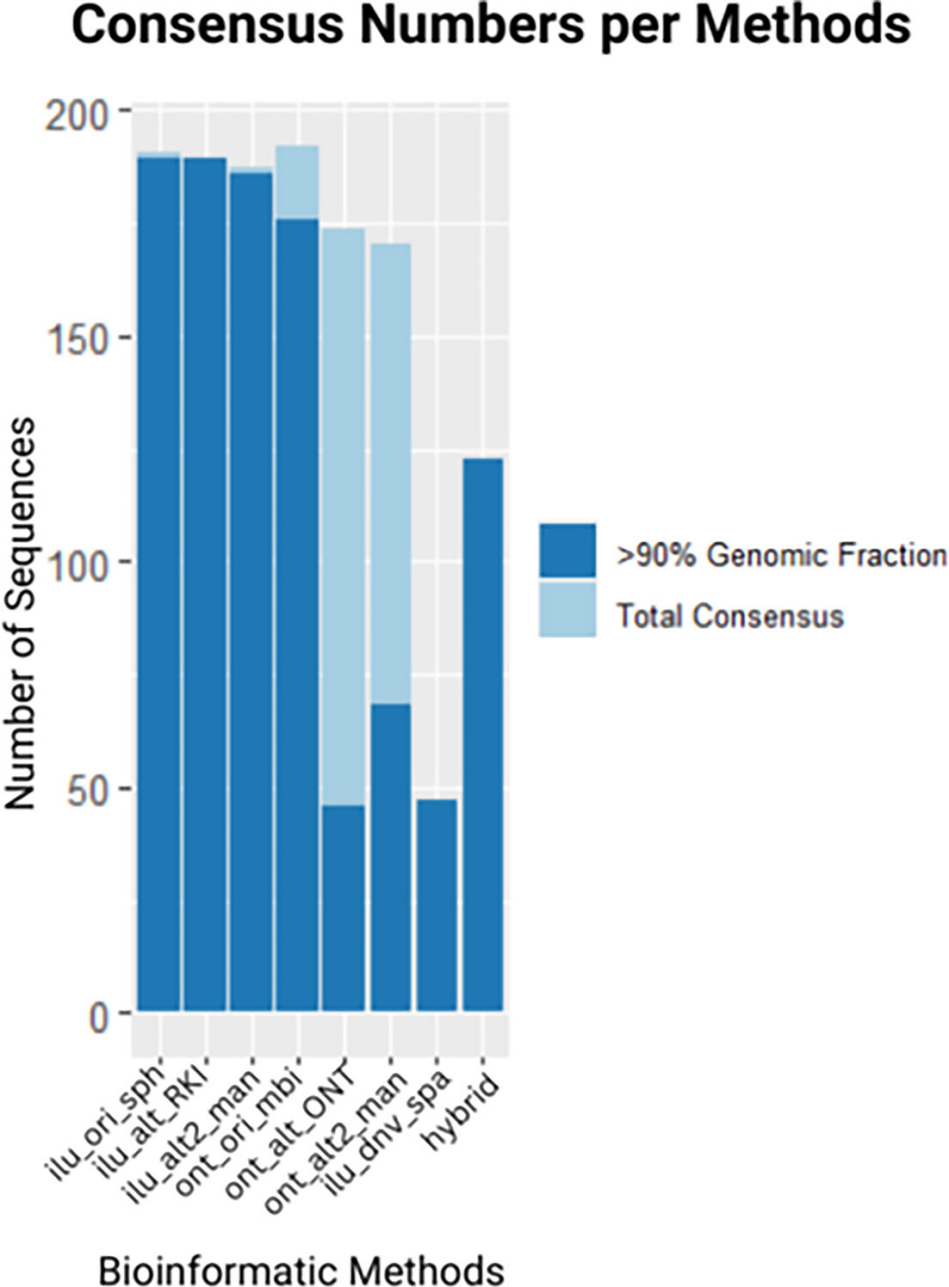
Overview of consensus sequence generation across bioinformatic methods: the bar chart compares the number of total consensus sequences (light blue bars) and those achieving >90% genomic fraction (dark blue bars) across different sequencing and assembly methods. The *x*-axis represents the bioinformatic methods utilized, while the *y*-axis indicates the number of sequences.

Despite earlier findings that some ONT data contained unclassified reads and non-viral sequences, the original ONT method performed best in generating complete consensus genomes, with most genomes exceeding 90% completeness (Fig. 3). Alternative ONT methods (ONT ALT and ONT ALT2) produced fewer complete genomes, though slight improvements were observed with the in-house method.

The HA method generated more identifiable consensus genomes than the Illumina *de novo* assembly and RKI pipeline. It identified 123 genomes, compared to 47 genomes identified by Illumina *de novo* assembly (Table 1). This represents over 2.5 times more genomes recovered by the hybrid method. Pipeline differences were also evident in genome completeness. The hybrid method recovered genomes with higher completeness rates than the alternative methods. Variability between pipelines in lineage assignment and genome recovery highlights the role of bioinformatic pipeline choice in determining analysis outcomes.

### Overall metric comparison among the eight different methods, including HA

**Fig. 4.**
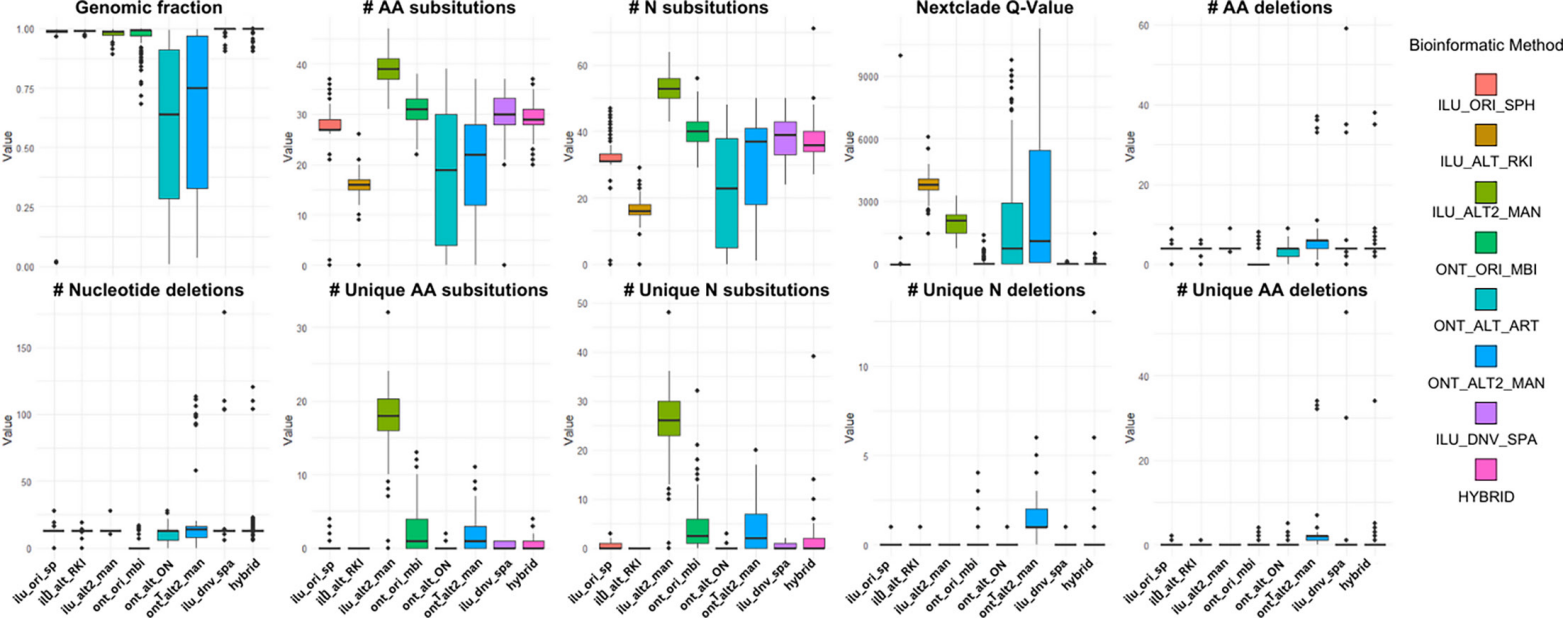
Comparative performance of consensus generation methods across various metrics: boxplots illustrate the distribution of values for eight bioinformatic methods across multiple sequencing metrics. Methods include SOPHiA DDM (ilu_ori_sph), CoVPipe (ilu_alt_RKI), in-house Illumina (ilu_alt2_man), nCOV analyser (ont_ori_mbi), ARTIC pipeline (ont_alt_ART), in-house ONT (ont_alt2_man), SPADes *de novo* assembly (ilu_dnv_spa) and hybridSPADES (hybrid). Metrics assessed are as follows: (**a**) genomic fraction, (**b**) amino acid (AA) substitutions, (**c**) nucleotide substitutions, (**d**) Nextclade quality metric, (**e**) AA deletions, (**f**) nucleotide deletions, (**g**) unique AA substitutions and (**h**) unique nucleotide substitutions. Each panel compares the performance of the methods in achieving the specified metric.

We compared the performance of eight methods across ten key genomic quality metrics, as illustrated in Fig. 4. The hybrid method demonstrated the highest genome fraction, defined as the number of aligned bases to the reference genome divided by the genome size. While this finding is statistically significant, the improvement is minimal, as the median genome fraction for Illumina-based methods, including *de novo* assembly, approaches 100%.

In terms of amino acid substitutions and deletions, the in-house methods for both Illumina and ONT platforms yielded higher values compared to proprietary and alternative methods. The in-house Illumina method detected the highest number of unique amino acid and nucleotide substitutions, while the in-house ONT method detected the highest number of deletions. Specific amino acid substitutions detected by the in-house Illumina method were unique to this approach, distinguishing it in this metric.

**Fig. 5.**
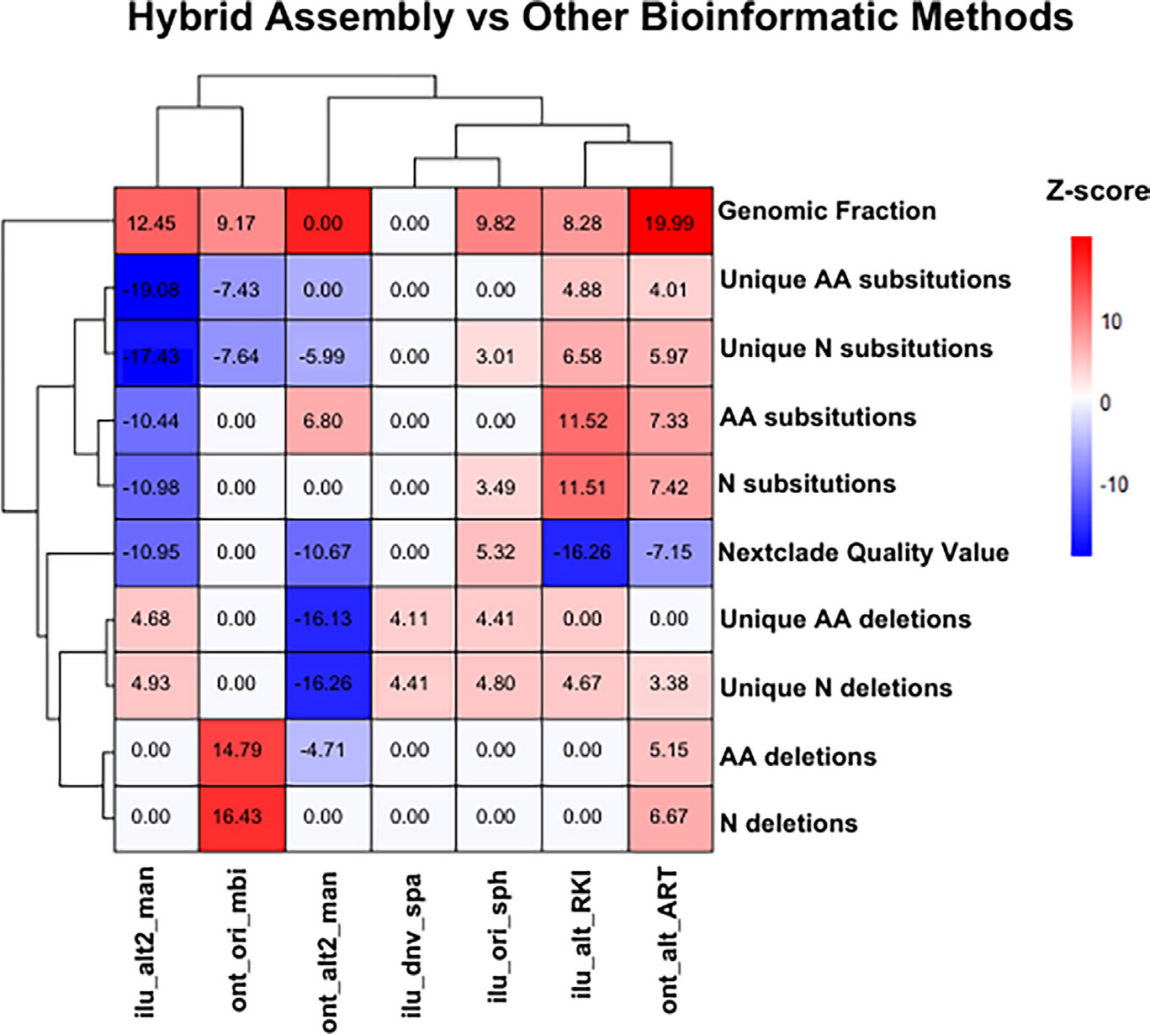
*Z*-score heatmap for comparative evaluation of the hybrid method: the heatmap presents *Z*-scores derived from Dunn’s test, comparing the hybrid method’s performance against other bioinformatic methods across various genomic metrics. Metrics include genomic fraction, amino acid (AA) substitutions, nucleotide substitutions, Nextclade quality value, AA deletions, nucleotide deletions and their unique variants. The colour scale represents the magnitude of the *Z*-scores, with red indicating higher *Z*-scores and blue indicating lower *Z*-scores. White cells indicate no significant difference between methods. Dendrograms show clustering of methods and metrics based on similarity.

Statistical evaluation using the Kruskal–Wallis test revealed significant differences (*P*<0.05) across all methods for all evaluated metrics. Pairwise comparisons using Dunn’s test revealed statistically significant differences in genome fraction between methods, as visualized in a *Z*-score heatmap (Fig. 5). The hybrid method achieved a statistically significant advantage in genome fraction but did not show notable differences in detecting unique amino acid substitutions, nucleotide substitutions or deletions.

Nextclade quality scores showed that the hybrid method produced genomes comparable to or superior to other ONT-based methods. However, the proprietary Illumina SOPHiA DDM method consistently yielded the highest genome quality across all evaluated metrics (Fig. S3).

### Mutational analysis for ‘high-confidence’ samples

To assess the performance of different methodologies for detecting amino acid (AA) substitutions, we constructed a consensus mutation dataset for 95 high-confidence samples out of the 192 total (described in the Methods section). This consensus served as a benchmark for assessing each method’s performance.

**Fig. 6.**
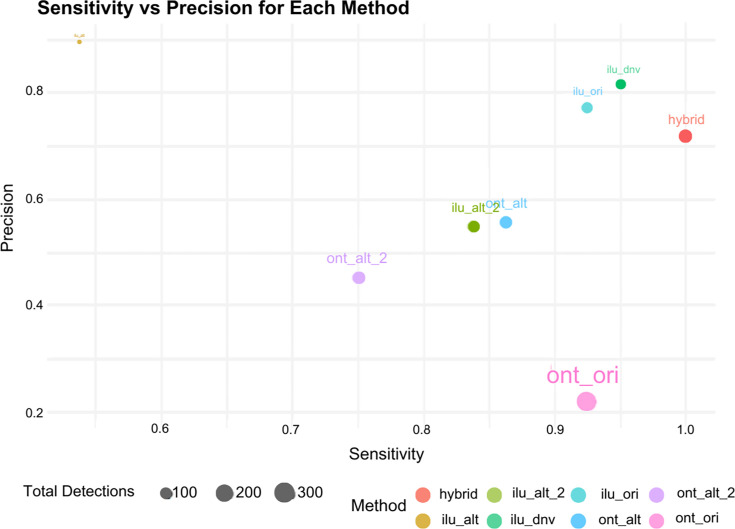
Sensitivity versus precision for amino acid substitution detection across methods. The scatter plot illustrates the relationship between sensitivity (*x*-axis) and precision (*y*-axis) for amino acid substitution detection by various bioinformatic methods. Sensitivity reflects the proportion of true mutations correctly identified, while precision represents the proportion of TP among all detected mutations. The size of each point corresponds to the total number of mutations detected by each method, providing an additional measure of detection capacity. Methods are colour-coded for clarity.

A sensitivity versus precision analysis, as shown in Fig. 6, revealed that the hybrid method achieved perfect sensitivity (1.0), successfully detecting all true mutations present in the consensus for the high-confidence samples. In contrast, the RKI pipeline (ilu_alt) exhibited the highest precision (0.90), indicating a strong likelihood that detected mutations were accurate, though it detected fewer mutations overall.

The proprietary ONT method (ont_ori) detected the highest number of mutations (~300) but exhibited lower precision (0.21), indicating a higher proportion of false positives. Fig. S3 confirmed that ONT-based methods, particularly ont_ori, detected the most unique mutations overall. However, mutations identified by the in-house Illumina method showed greater consistency across samples, while those detected by ont_ori were more sample-specific.

The hybrid method demonstrated a precision score of 0.72 alongside perfect sensitivity, making it effective in detecting a broad range of mutations, including those with low frequency. Further analysis of mutation detection frequencies revealed that the hybrid method detected comparable levels of high-, universal- and medium-commonality mutations compared to the proprietary Illumina pipeline. Additionally, it outperformed other methods in detecting lower-frequency mutations.

### Maximum-likelihood tree on 12 hybrid and method-specific representative sequences

**Fig. 7.**
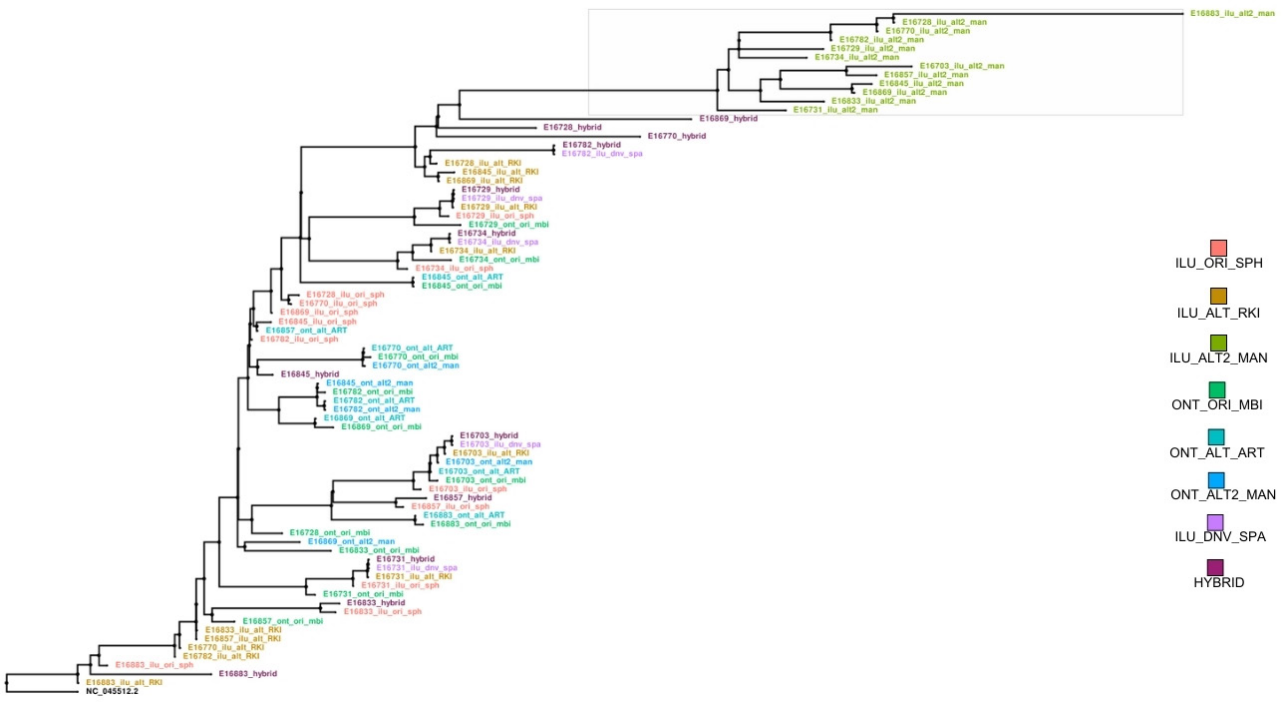
Maximum-likelihood phylogenetic tree of representative SARS-CoV-2 sequences. The phylogenetic tree, rooted in the SARS-CoV-2 reference genome (NC_045512.2), depicts the genetic relationships among representative sequences produced by various sequencing and assembly methods. Branches are colour-coded to indicate the corresponding bioinformatic method, highlighting differences in sequence clustering and relatedness. The tree provides an overview of method-specific variation in consensus sequence generation.

A maximum-likelihood phylogenetic tree rooted in the reference genome was constructed to evaluate potential biases and sequencing artefacts across methods. This analysis included 12 representative sequences from the hybrid dataset (*n*=123), ensuring all sequences were generated using the HA. Branch colours distinguished consensus sequences by method, enabling detection of method-specific clustering (Fig. 7).

The hybrid method’s sequences were evenly distributed throughout the phylogenetic tree, with no evidence of clustering observed (Fig. 7). Sequences generated by the hybrid method showed no signs of method-specific artefacts, suggesting impartiality in sequence generation.

Sequences generated by the in-house Illumina method clustered in the upper portion of the phylogenetic tree, indicating potential method-specific biases. This clustering could be associated with sequencing artefacts that may introduce non-existent mutations into consensus sequences. Proprietary ONT and Illumina pipelines did not exhibit similar clustering patterns, suggesting a lower likelihood of sequencing artefacts associated with these methods.

### Understanding sample conditions to employ HA for SARS-CoV-2

**Fig. 8.**
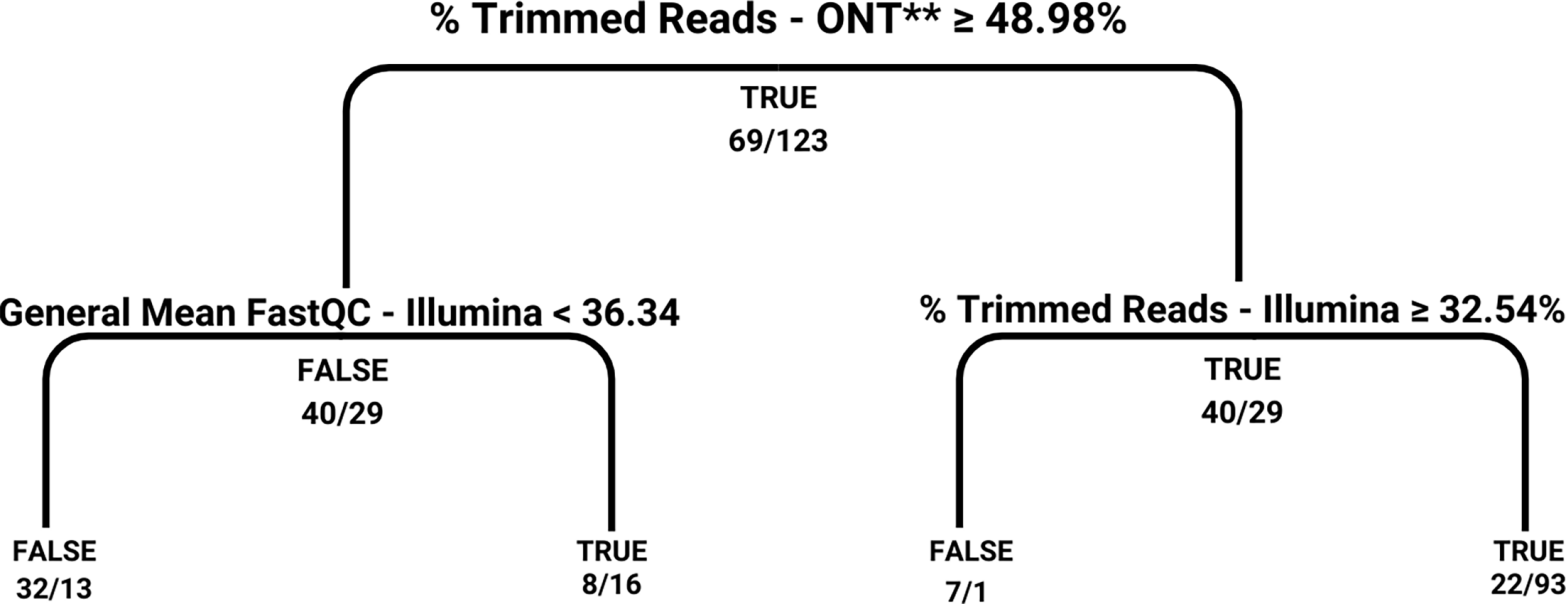
Decision tree for predicting HA success. The classification tree demonstrates the hierarchical decision-making process for determining the likelihood of successful HA. The model uses thresholds for the percentage of trimmed reads from ONT and Illumina sequencing, as well as the general FastQC scores for Illumina. Each branch represents a decision criterion, with outcomes (TRUE/FALSE) and the corresponding sample counts displayed at each node.

To identify the conditions for optimal hybrid sequencing of SARS-CoV-2, logistic regression and decision-tree classification models were applied to ten sample properties derived from Illumina and ONT platforms. These models evaluated the probability of successful HA (Fig. 8).

The decision-tree analysis identified the percentage of trimmed reads from ONT as a critical predictor of assembly success. Samples with trimmed read percentages at or above the 48.98% threshold were consistently associated with successful hybrid assemblies. This result highlights the role of high-quality ONT reads in enabling effective HA for SARS-CoV-2 genomic reconstruction.

Illumina FastQC scores below 36.34 increased the likelihood of false negatives. However, lower scores did not preclude successful HA, indicating that HA can be effective even with lower-quality Illumina data, provided high-quality ONT data are available. Conversely, Illumina FastQC scores exceeding 36.34 were associated with overpredictions of assembly success, suggesting that Illumina data quality alone is insufficient as a reliable predictor.

These findings indicate that combining high-quality ONT data with Illumina data improves assembly outcomes. The decisive role of ONT read quality suggests it could serve as a key criterion for determining when to employ hybrid sequencing strategies for SARS-CoV-2.

## Discussion

This study evaluated the conditions under which HA enhances the generation of SARS-CoV-2 consensus genome sequences, comparing results from Illumina MiSeq and ONT GridION platforms. Additionally, it assessed the performance of short- and long-read sequencing methodologies for the same purpose. Our findings reveal that while HA does not universally outperform single-platform methodologies in mutation detection, it reliably identifies mutations and improves genome fraction coverage compared to both platform-specific approaches. This underscores its utility as a validation and improvement tool in public health SARS-CoV-2 surveillance, especially when dealing with low-quality, high-priority samples.

Our regression analysis identified ONT read trimming as a significant predictor of HA success. Higher levels of trimming, often associated with lower-quality samples, correlated with successful assemblies, indicating that high-quality ONT reads are conducive to effective HA. However, the utility of read trimming as a predictor remains limited, as it indirectly indicates data quality. Notably, this is the first study to report the influence of ONT read trimming on routine SARS-CoV-2 sequencing, highlighting its importance in data recovery from low-quality samples. Understanding predictors of HA success is essential for maximizing its potential in genomic surveillance.

In terms of genome fraction coverage, the hybrid method showed a statistically significant but minimal increase compared to Illumina-only data, aligning with previous research. Studies such as those by Kouriba *et al.* [31,33, 69] have employed HA to enhance genome fractions in SARS-CoV-2 WGS and low-coverage SARS-CoV-2 WGS. Our findings extend this knowledge, demonstrating that HA improves genome fraction even in high-coverage samples (over 95%) while also outperforming alternate ONT pipelines in generating complete genomes. This suggests that HA is useful for low-coverage samples and can also act as a validation tool for Illumina sequencing, especially when unexpected results arise.

Interestingly, the proprietary ONT pipeline performed well despite lower SARS-CoV-2 reads compared to the paired Illumina reads, contrasting with the alternate ONT pipelines, which underperformed. This variability emphasizes the importance of in-house validation of sequencing results, especially for new or adapted methodologies. HA also proved valuable for validating ONT sequencing outcomes, enhancing accuracy and optimizing the management of sequencing resources in labs with access to both platforms.

Our exploration of the Illumina in-house method uncovered artificial bias introduced by untrimmed sequencing primers, which negatively impacted the performance of other methods, such as the RKI pipeline. This reinforces the need for best practices in SARS-CoV-2 bioinformatic analysis, as discussed in existing literature [70,72]. The clustering of sequences due to these biases underscores the importance of validation checks to ensure data accuracy.

While proprietary pipelines like Illumina’s generate high-quality consensus genomes, they also pose challenges for public health genomics due to restricted access to the necessary information for developing independent pipelines. This limitation risks creating dependence on commercial solutions. To mitigate this, public health institutions should adopt open and transparent methodologies that allow for greater autonomy in genomic surveillance. This would also address the bioinformatics expertise gap in many public health settings.

Despite using identical nucleic acid extracts, we also observed a higher proportion of non-SARS-CoV-2 reads in ONT-sequenced samples compared to Illumina. After applying a read-length filter, SARS-CoV-2 reads were further reduced, likely because the virus-derived reads were shorter. Although RNA levels are typically unaffected by multiple freeze-thaw cycles in qPCR experiments [73], it is possible that amplicon generation in our study was impacted, leading to shorter reads. Our investigation of unclassified reads also identified a significant proportion of unnatural sequences, such as barcodes. While trimming reduced the number of these sequences, SARS-CoV-2 motifs were still detected, suggesting potential contamination due to the Rapid Barcoding Kit, which may have introduced chimeric nonsense reads.

The emergency conditions under which many COVID-19 sequencing efforts occurred in Türkiye resulted in necessary good practices being overlooked during sequences, as the incentive was to sequence as many SARS-CoV-2 samples in the shortest period of time. While NGS best practices exist for clinical microbiology [74,75] and public health laboratories [76], the need for standardized protocols is especially pressing in newly established laboratories that conduct routine sequencing. This need is underscored by the absence of key metrics, such as *Cq* values and input concentrations, in our study.

In the absence of these metrics, we sought to understand the nature of our data and the impact of bioinformatics methods by using SARS-CoV-2-specific read numbers as a surrogate measure. While not intended as a comparative metric, this approach highlighted platform-specific biases, such as ONT producing significantly fewer SARS-CoV-2 reads than Illumina. To mitigate these biases, we also examined SARS-CoV-2 read percentages, which normalized read output but remained influenced by platform-specific factors like sequencing chemistry and bioinformatics pipelines. These challenges emphasize the importance of robust experimental measures to complement computational analyses.

Quality scores, although differing significantly between platforms due to distinct derivation methods, were used to evaluate platform-specific performance trends, following previous studies [77]. Our analysis confirmed that ONT base quality scores, though lower on average, did not indicate any inherent issues with the quality of ONT data, ruling out base quality as a cause of discrepancies in SARS-CoV-2 read numbers. Instead, platform-specific biases in technology and workflow were implicated.

The absence of *Cq* values and controls during sequencing further limited the ability to assess RNA quality and pre-library amplification efficiency. These findings highlight the need for systematic improvements in laboratory workflows, particularly in emergency settings. Türkiye’s development of a national genomic surveillance strategy, aligned with WHO guidelines, aims to address these gaps by standardizing practices and enhancing sequencing reliability even in challenging conditions.

While hybrid methods are promising, their routine application may be impractical due to the resource-intensive nature of hybrid sequencing and the complexity of integrating different platforms. However, HA remains valuable for specific high-priority cases, such as those requiring high-accuracy ONT sequencing results. Future research should focus on streamlining hybrid methodologies to make them more feasible for routine use.

Recent improvements to ONT sequencing, such as adaptive sampling (AS), R.10 flow cells and new basecalling models, can improve the quality of sequences and reduce the need for HA. AS has been shown to enrich SARS-CoV-2 amplicons successfully [78], which could be particularly useful for low-quality, high-value samples. Although not used in this study, implementing R.10 chemistry and the SUP basecalling model may further enhance sequencing quality [79,80]. Nevertheless, the HA could still improve results in such cases.

This study contributes to understanding genomic surveillance methodologies in the context of the COVID-19 pandemic. Our findings underscore the potential of hybrid sequencing approaches to enhance the accuracy and reliability of genomic data. Future research should aim to refine these methodologies, address their practical limitations and explore their broader applications in public health surveillance.

## Supplementary material

10.1099/mgen.0.001357 Fig. S1.

10.1099/mgen.0.001357 Table S1.

## Abbreviations

AS: adaptive sampling
HA: hybrid assembly
NGS: next-generation sequencing
NMMRL: National Molecular Microbiology Reference Laboratories
NVRL: National Reference Virology
ONT: Oxford Nanopore Technologies
OOB: out-of-bag
PHEIC: public health emergency of international concern
qPCR: quantitative PCR
SARS-CoV-2: severe acute respiratory syndrome coronavirus 2
SNPs: single nucleotide polymorphisms
VOC: variants of concern
VOIs: variants of interest
WCSS: within-cluster sum of squares
WGS: whole-genome sequencing
