# Supplementary material for: Enhancing public health surveillance: a comparative study of platform-specific and hybrid assembly approaches in SARS-CoV-2 genome sequencing
Source: Microb Genom. 2025 Jul 10;11(7):001357. doi: 10.1099/mgen.0.001357 (PMC12244368; doi:10.1099/mgen.0.001357)
Supplement: Fig. S1. [file mgen-11-01357-s001.pdf]

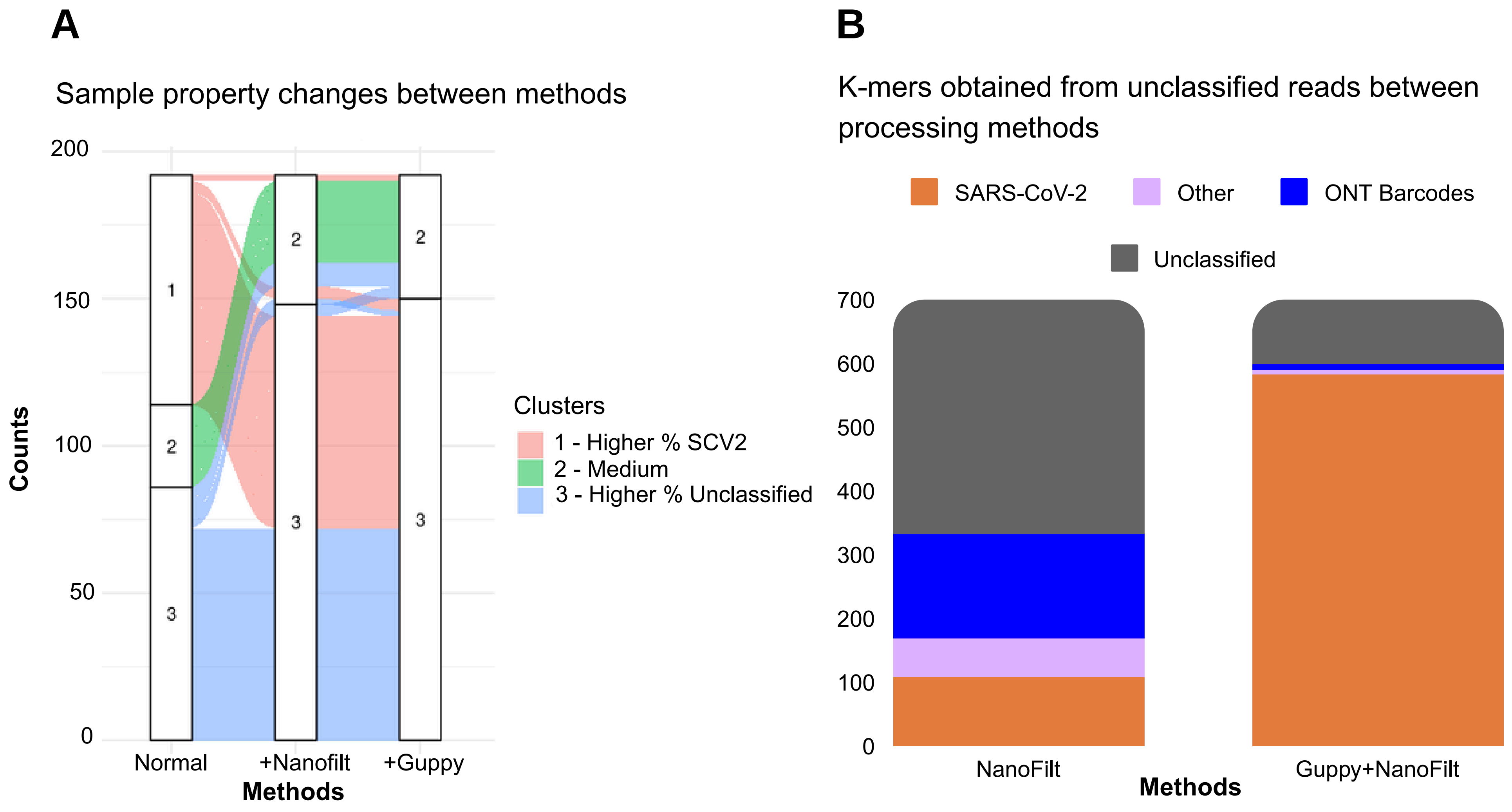

Supplementary Figure 1: Effect of processing methods on ONT reads. A) Changes in sample composition as processing steps are introduced. As introduction of sample processing methods the amount of SARS-CoV-2 classified reads decrease and samples tend to have proportionally more unclassified reads. **B)** The top 700 k-mers from unclassified reads were classified, between samples that have been just filtered with NanoFilt (left bar) and those that have undergone Guppy barcode trimming.

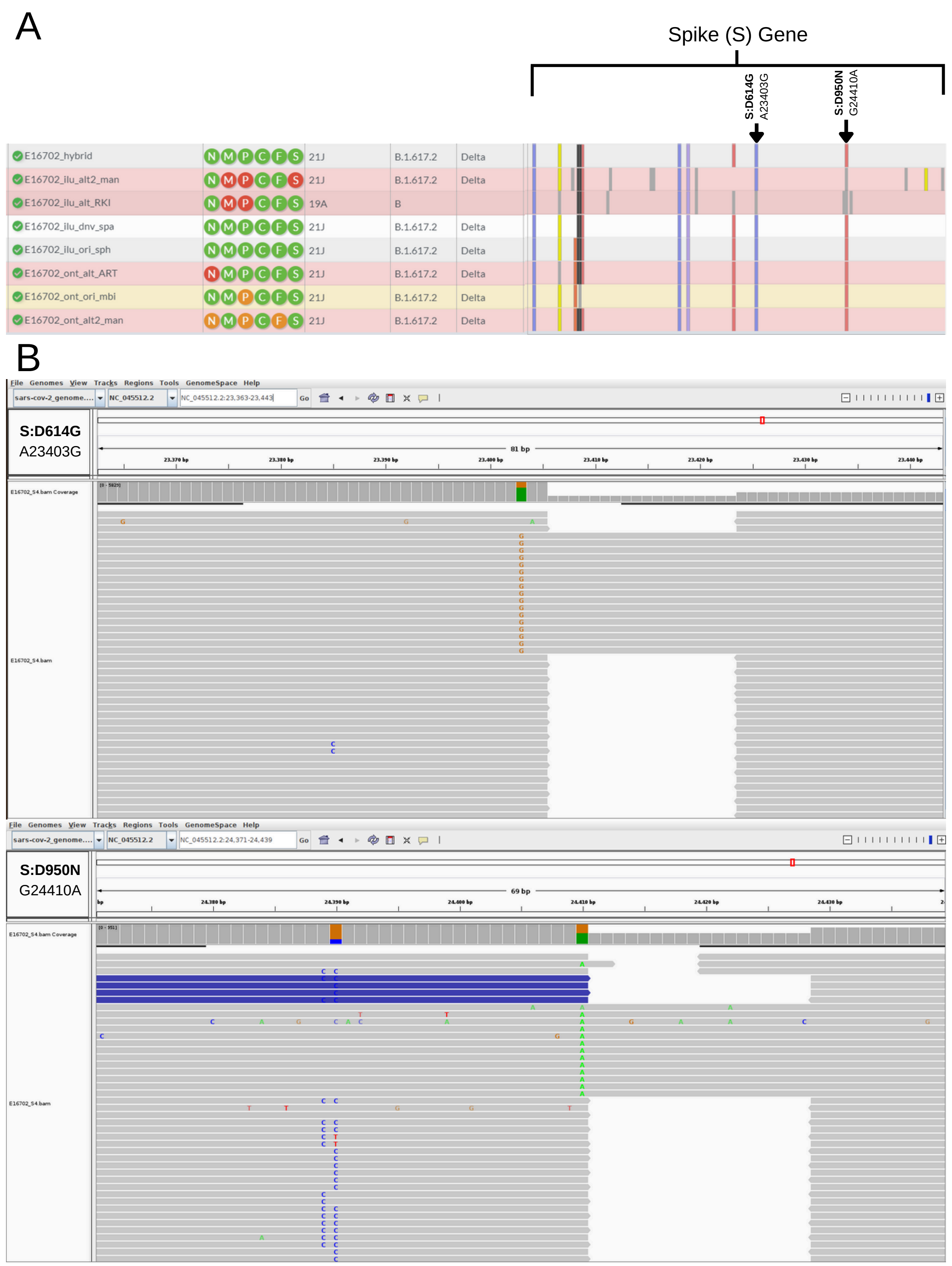

Supplementary Figure 2: Ambiguous nucleotide assignments are present in the sample with primer sequences. A) Nextclade image of sample E16702, with all eight methods consensus sequences. The third row of the RKI pipeline (ilu_alt_RKI) shows ambiguous bases in locations A23403G and G24410A. **B)** Image from the Integrated Genomics Viewer (IGV) shows conflicting images in both nucleotide positions.

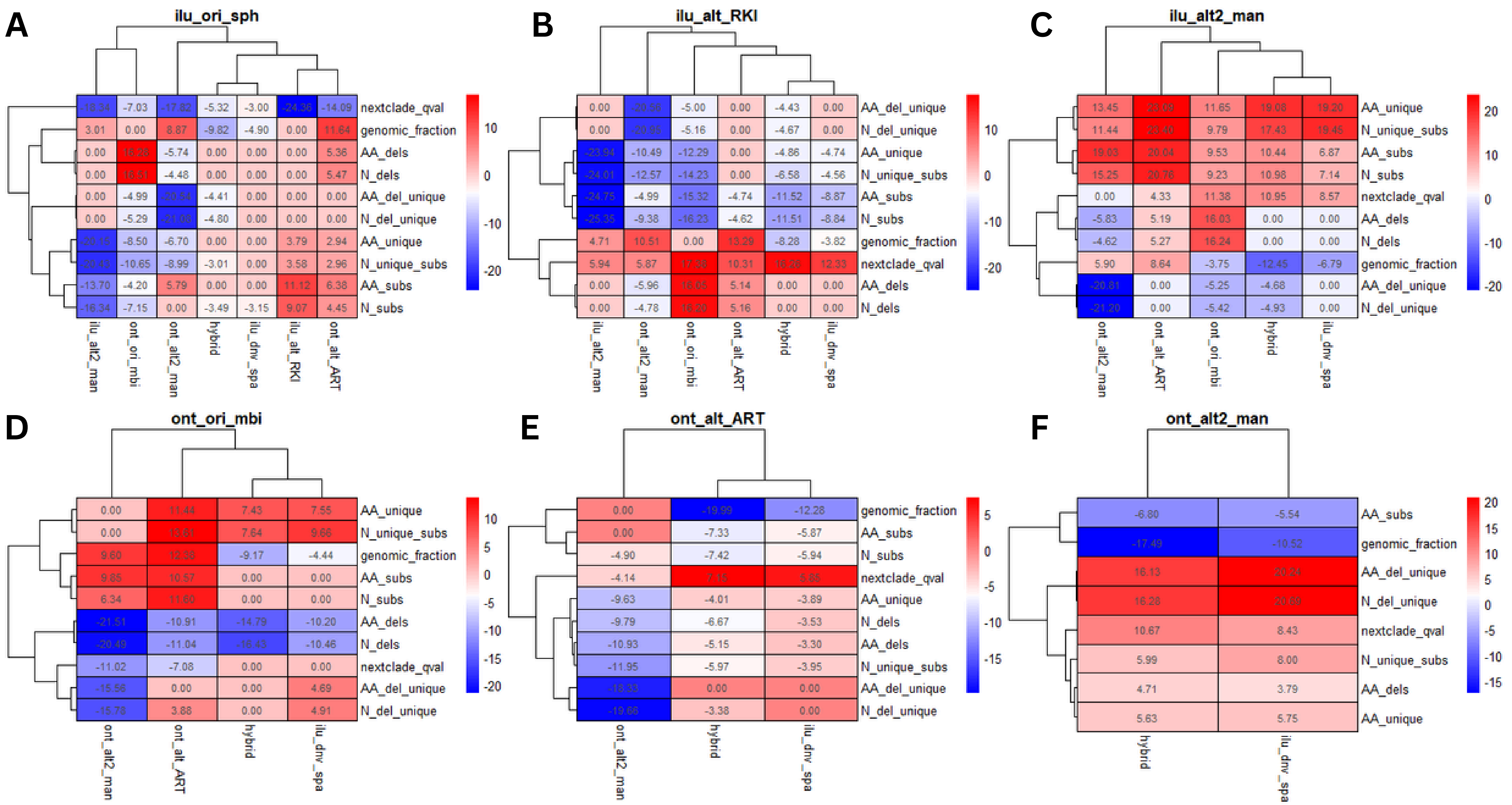

Supplementary Figure 3: Z-Score Heatmaps for Various Bioinformatic Approaches. The heatmaps display Z-scores from Dunn's test, comparing the method in the panel title with the methods listed on the X-axis. Each panel (A to F) represents comparisons for a different bioinformatic method: SOPHiA DDM, CoVPipe, in-house Illumina, ncov analyzer, ARTIC pipeline, and in-house ONT methods, respectively. The number of methods on the X-axis decreases in successive panels as comparisons are limited to the remaining methods not included in previous panels. Red shading indicates higher Z-scores, while blue shading represents lower (negative) Z-scores.

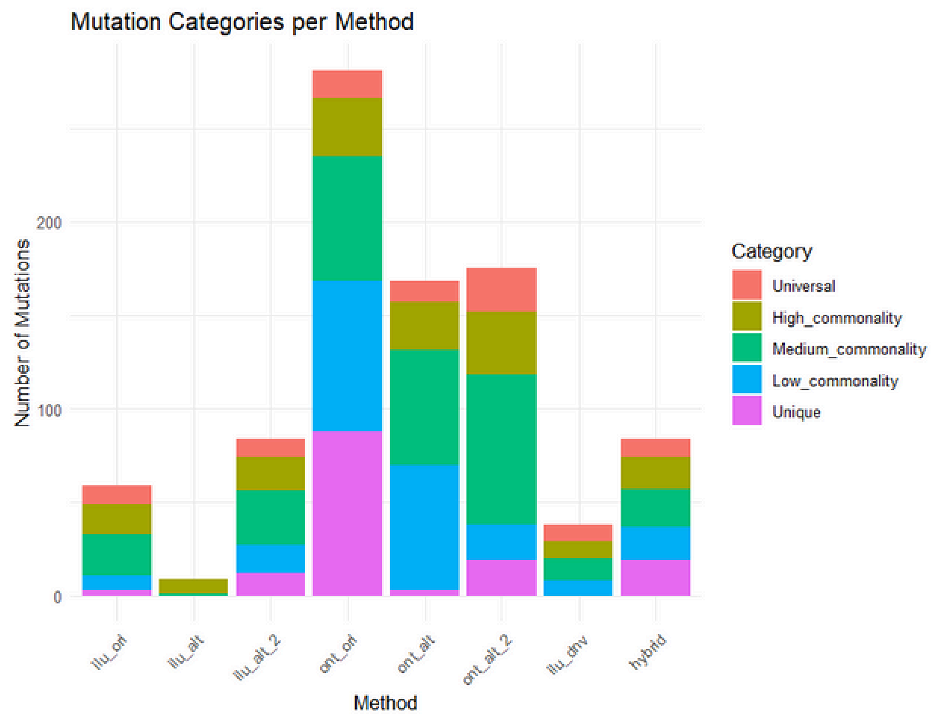

Supplementary Figure 4: Mutation Categories Detected by Each Method in High-Confidence Samples. This bar plot categorizes mutations based on their detection commonality across eight sequencing methods. Categories range from 'Unique', indicating mutations identified by only one method, to 'Universal', representing mutations detected by all methods. Intermediate categories include 'High commonality' for mutations found in six or seven methods, 'Medium commonality' for those observed in four or five methods, and 'Low commonality' for mutations noted in two or three methods. Each bar's height reflects the total number of mutations detected within each category, providing insight into the comparative mutation detection capabilities of each method.

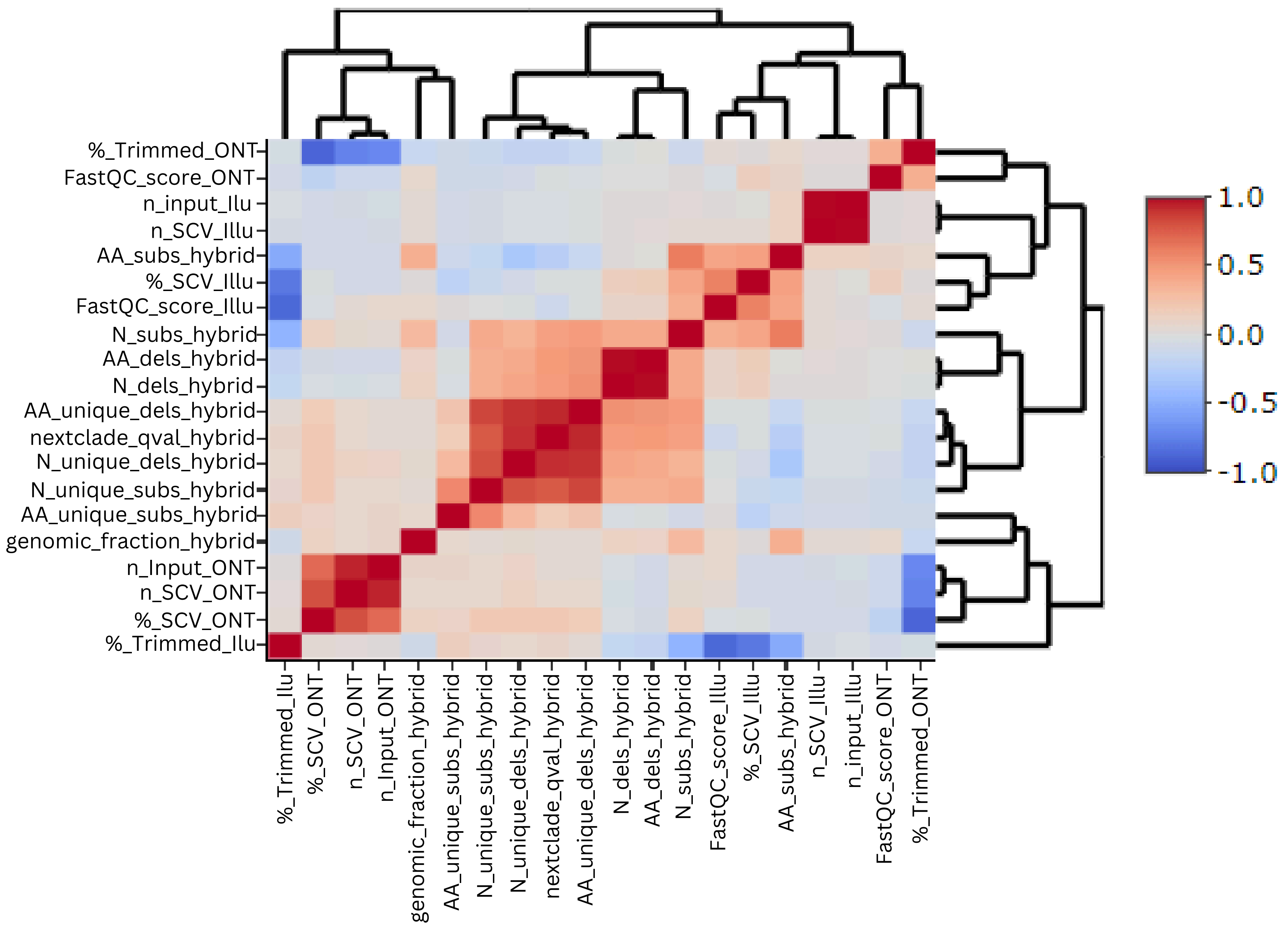

Supplementary Figure 5: Correlation Matrix of sample properties in hybrid assembly. The heatmap shows pairwise correlations between metrics derived from the hybrid bioinformatic pipeline and platform-specific sample metrics (ONT and Illumina). The X-axis and Y-axis list metrics, hierarchically clustered to group related features based on their correlations. Metrics on the X-axis include properties specific to hybrid assemblies, while platform metrics (identical across ONT or Illumina approaches) are included for comparison. The color scale indicates correlation strength, with red representing strong positive correlations and blue representing strong negative correlations. Hierarchical clustering was applied to reveal patterns in metric relationships, helping to identify dependencies between bioinformatic pipelines and platform-specific properties.
